# Estimating population norms for the health-related quality of life of adults in southern Jiangsu Province, China

**DOI:** 10.1038/s41598-022-13910-x

**Published:** 2022-06-14

**Authors:** Wei Hu, Liang Zhou, Jiadong Chu, Na Sun, Xuanli Chen, Siyuan Liu, Yueping Shen

**Affiliations:** 1 Department of Epidemiology and Biostatistics, School of Public Health, Medical College of Soochow University, 199 Renai Road, Suzhou, 215123 People’s Republic of China; 2 Liyang Center for Disease Control and Prevention, 55 Nanhuan Road, Liyang, 213371 People’s Republic of China

**Keywords:** Health care, Health occupations

## Abstract

Although national health-related quality of life population norms had been published based on the EuroQol 5-Dimensions 5-levels scale, China is a vast country with diverse cultural and social development in various regions. Therefore, regional population norms may better reflect the health status of residents in a given area. The purpose of the study was to derive the HRQoL population norm for adult general population in southern Jiangsu Province using the EQ-5D-5L scale and explore potential influencing factors. The data were based on a cross-sectional survey conducted in Liyang City from March 2019 to July 2020. EQ-5D-5L utility scores based on Chinese value set and EQ-VAS scores were used to assess HRQoL. The Tobit regression model and generalized linear model were performed to identify the association among potential covariates and HRQoL. The means (95% confidence interval) of the EQ-5D-5L utility scores and EQ-VAS scores were 0.981(0.980–0.983) and 83.6(83.2–83.9), respectively. Younger people (≤ 40 years old) were more likely to experience problems with anxiety or depression. Additionally, women had lower HRQoL scores although multivariate analysis found no statistical difference between the sexes. Lower HRQoL was associated with advanced age, lower socioeconomic status, no spouse, lack of regular physical activities, smoking cessation, and chronic non-communicable diseases. Subjects who declared that they were afflicted by diseases presented significantly lower utility scores, ranging from 0.823 (0.766–0.880) for memory-related diseases to 0.978 (0.967–0.989) for hepatic diseases. Regional population norms of HRQoL are needed in the health economic study owing to the great socioeconomic differences across regions in China. The present study provides HRQoL population norms for adults in southern Jiangsu. These norm values could help policy makers better allocate limited health resources and prioritize service plans.

## Introduction

In recent years, economic evaluation has been widely adopted to guide clinical practice and public health policy decisions^1^. One of the most frequently reported benefit measurements in economic evaluations is quality-adjusted life years, which combines the quality of life and length of life into a single health-related quality of life (HRQoL) score^2^. If a study does not have a control group and wants to evaluate the effect of its intervention, an estimate for HRQoL utility score norms in the general population can serve as a reference group^2^. Moreover, population norms enable researchers to compare profiles of patients with particular diseases with data on the health status of the general population of similar age and sex and measure health inequalities^3^. HRQoL population norms can also be treated as the key to determining whether the scores of a specific group or individual are above or below the average level for a similar population^4^. Therefore, assessing the HRQoL of the general population to construct population norms is helpful for long-term monitoring of health status, determining groups with poor HRQoL, assessing the burden of diseases on HRQoL, and exploring the relationship between different sociodemographic factors and health status^5^ and ultimately optimizing the efficiency of medical resource allocation. Therefore, it is becoming an inevitable requirement for the development of healthcare in each country.

HRQoL is a self-reported outcome that is increasingly used to monitor the health status of the general population. It is a multidimensional concept that reflects the physical, psychological, social and emotional well-being of the respondents^6^. To date, there are many different instruments for measuring HRQoL, including the EuroQol five-dimension (EQ-5D), Health Utilities Index (HUI)^7^, and the Short -form 6-dimension (SF-6D)^8^. The EQ-5D is one of the major self-reported instruments to evaluate HRQoL due to its simplicity, low respondent burden and high universal acceptance^9,10^. There are two versions of the EQ-5D, namely, the EQ-5D-3L and the EQ-5D-5L. In recent studies, the EQ-5D-5L has been widely used because it can reduce the ceiling effect (the proportion of respondents reporting “no problems” for all dimensions) detected in the EQ-5D-3L^11^. This method has higher convergence validity and is more sensitive to slight health changes^12,13^. Additionally, an existing EQ-5D-5L value set from the Chinese adult general population is now available^14^. In a given region of China (e.g., southern Jiangsu), the study on the evaluation of HRQoL in the general population by the EQ-5D-5L scale is still quite limited. Therefore, this EQ-5D-5L scale was used to evaluate the quality of life of the respondents in this study.

In the field of public health, the population norm (average population score) of a region is often expressed as the EQ-5Dutility score^15^, and the population norm is the benchmark for evaluating population health care and health equity. Although the EQ-5D-5L population norm has been reported in China's urban population^16^, due to its small sample size (1332 people were included in 5 regions), it may not be well representative of the rest of China, let alone southern Jiangsu, which has a relatively developed economic level. The southern region of Jiangsu Province (including Nanjing, Suzhou, Wuxi, Changzhou, Zhenjiang) is located in the centre of the Yangtze River Delta along the southeast coast of China, with over 30 million permanent residents^17^. All counties (cities) have entered the ranks of the top 100 counties of national comprehensive strength, of which 7 counties (cities) have entered the top 10, which is one of the most developed and modernized regions in China. Therefore, the HRQoL norm in this region can provide a reference for the health level of the population in most developed regions of China. Furthermore, China is a vast country, and the cultural and social development of each province is different^18^. Thus, regional population norms may better reflect the health status of residents in a given area. The main purpose of this study is to derive the HRQoL population norms in the southern area of Jiangsu Province, China based on a large number of randomly sampled data from the Liyang Chronic Disease Risk Factor Monitoring Cohort Study (The Liyang Study) and the EQ-5D-5L China value set. Second, we examine the association between sociodemographic characteristics, health needs and HRQoL.

## Methods

### Study design and data collection

The Liyang Study is a face-to-face cross-sectional observational study conducted between March 2019 and June 2020. Liyang is located in the south of Jiangsu Province, with a permanent, year-round population of approximate 0.8 million in 2019. As one of the top 100 counties (top 100 in comprehensive strength of all counties, county-level cities and some municipal districts in China) in China (No. 35), Liyang lies at the crossroads of the Nanjing metropolitan area and Shanghai economic zone. Therefore, the HRQoL status of the general population in Liyang can reflect the basic situation of the population in southern Jiangsu to a certain extent. The method of multi-stage cluster random sampling was adopted to randomly select 17 health centres in 12 towns of Liyang City for investigation (Supplementary Fig. 1). We designed a standardized questionnaire including sociodemographic characteristics, behavioural lifestyle factors, health-related information, and the EQ-5D scale to collect data.

We preliminarily planned to conduct a large-scale survey covering ten thousand people among the general population of Liyang City. First, to ensure a sufficient sample size and facilitate implementation and calculation, a random sample of approximately 600 cases from each of the 17 health centres was planned for the survey. However, the actual final sample size varied from 443 to 633 per site (an average of 600 cases per health centre) for some reason, such as the absence of an address, some residents refusing to be surveyed or being physically unable to be surveyed (Supplementary Table 1). Furthermore, two administrative villages or communities closest to each health centre were selected and approximately 300 cases were randomly selected from each village or community as the investigation objective. Then, under the auspices of the local CDC, randomly selected residents were asked to go to the nearest health centre (or community hospital) for an investigation. Next, trained local health workers were recruited to conduct a questionnaire survey for each selected participant through face-to-face interviews at each health centre, and the interviews were recorded for subsequent quality control. The questionnaires were issued and retrieved on the same day, and missing or incorrect items were supplemented, proofread and logically corrected on time. The inclusion criteria were as follows: local permanent residents (living ≥ 6 months in local before investigation), age ≥ 18 years, complete sociodemographic information, and completion of the EQ-5D-5L scale. Before the study began, participants were informed about the purpose of the study, its health benefits and potential harms. Participants were asked to give written informed consent, and both researchers and respondents agreed to use the data only for scientific research purposes. This study was approved by the Ethics Review Committee of Soochow University and all respondents provided written informed consent. All methods were performed in accordance with the relevant guidelines and regulations.

### EQ-5D-5L

The EQ-5D-5L scale for the determination of the population HRQoL consists of a five-dimensional health description system and a self-reported overall health score using the EuroQol Visual Analog Scale (EQ-VAS). The descriptive system comprises the dimensions of mobility (MO), self-care (SC), usual activities (UA), pain/discomfort (PD), and anxiety/depression (AD), and each dimension has five levels of response (from “no problems” to “extreme problems”)^19^. To better understand the distribution of health problems experienced by respondents, we converted each dimension into the dichotomous variables of "0"(no problems) and "1"(problems, slight/moderate/severe/extreme problems are collectively referred to as “problems”). The utility score is generated by applying the Chinese time trade-off model developed by Liu et al.^20^. The utility score ranges from − 0.391 for the worst (55,555) to 1 for the best (11,111) EQ-5D-5L health states. The EQ-VAS score is the self-reported overall health perception of the respondents. It records the respondent’s self-rated health on a vertical scale from 0 (the worst health) to 100 (the best health), where the respondents picture their health status on the interview day. Respondents with high utility scores or VAS scores had a better HRQoL than those with low scores.

### Ethical approval

This study was approved by the Ethics Review Committee of Soochow University and all respondents provided written informed consent.

### Consent to participate

All respondents provided written informed consent prior to the interview.

## Covariates

### Sociodemographic factors

Sociodemographic factors including sex, age (18–30, 31–40, 41–50, 51–60, 61–70, and 70 + years), residence(urban/rural), education level (primary school and below, junior middle school, high school or similar and junior college and above), marital status(married, unmarried/divorced/widowed), annual household income (< 50,000 yuan, 50,000–99,999 yuan, 10,0000–14,9999, and ≥ 15,0000 yuan, RMB), employment status(employed/full-time/part-time, unemployment/retirement/housework/student), and body mass index(BMI, low BMI, < 18.5 kg/m^2^; normal BMI, between 18.5 and 24.0 kg/m^2^; overweight BMI, between 24.0 and 28.0 kg/m^2^; and obese BMI, ≥ 28.0 kg/m^2^, following recommendations from the Working Groupon Obesity in China^21^).

### Behavioural lifestyle factors

Behavioural variables included: smoking, defined as at least one cigarette per day for 6 months (never, current, or former); alcohol consumption, defined as at least once a month (never, current); and regular physical activity, defined as moderately intense activity lasting more than 10 min at least once a week (yes, no)^22^.

### Health-related factors

The participants were also asked whether they had a health problem affecting HRQoL (e.g., mental illness, memory-related illness), and their general health needs. Health needs were measured by chronic non-communicable diseases (NCDs), which included hypertension, diabetes, dyslipidemia, stroke, coronary heart disease (CHD), asthma, chronic obstructive pulmonary disease (COPD) and cancer. NCDs were defined as a condition diagnosed by a doctor from a secondary or above medical institution, for which either the symptoms persisted or relevant medical treatment continued over the last 12 months^23^. Participants were classified as having no NCDs, or one, two or more NCDs.

### Statistical analysis

Frequencies and percentages were used for categorical variables, and all covariates including age were entered into the model as categorical variables. Means and 95% confidence intervals [CIs] were calculated for the continuous variables (including the EQ-5D-5L utility score and EQ-VAS score). The differences in utility scores and VAS scores between different subgroups were tested by employing Wilcoxon or Kruskal–Wallis tests. The Wilcoxon test was also used to describe differences in utility scores and EQ-VAS scores between men and women for age categories, education levels, income levels, and BMI categories. To deeply understand the impact of health restrictions in each EQ-5D dimension on HRQoL, we converted each dimension of the EQ-5D scale into binary variables with or without health problems, and then calculated the mean utility scores and VAS scores of respondents with or without health problems in each dimension respectively. Mann–Whitney test was used to examine the difference in the mean utility score and VAS score in different dimensions of the EQ-5D-5L scale. In addition, the chi-square test was used to compare the incidence of problems for each EQ-5D dimension by gender and age.

The relationships between all covariates and EQ-VAS scores were explored using a generalized linear model (GLM) with a Poisson distribution and a log link based on the modified Park test^24^. Since the distribution of the EQ-5D utility scores was skewed and censored at 1, we used the multivariate Tobit regression model to evaluate the relationship between the EQ-5D utility score and potential influencing factors^25^. All data analysis was performed in SAS version 9.4(SAS Institute Inc., Cary, NC, USA) and STATA version 15.0. A *P* value below 0.05 was considered statistically significant.

## Results

### Sample characteristics

A total of 10,200 individuals aged 18 and above participated in the study, 144 of whom were excluded because they did not meet the inclusion criteria, leaving 10,056(response rate: 98.6%) respondents who enrolled in this study. All respondents completed the five dimension sections of the EQ-5D-5L scale, but only 5080(53.8%) respondents completed the EQ-VAS score section. The sociodemographic characteristics and health needs of the respondents are summarized in Table 1.

**Table 1:** EQ-5D-5L utility scores according to sample characteristics.

Subject characteristics		EQ-5D-5L utility scores	
	n (%)	Mean (95%CI)	*P*
Overall	10,056	0.981 (0.980–0.983)	
**Sex**			
Male	4734 (47.08)	0.983 (0.981–0.985)	0.001**
Female	5322 (52.92)	0.980 (0.978–0.982)	
**Age group (year)**			
18–30	1407 (13.99)	0.996 (0.994–0.998)	< 0.001**
31–40	1699 (16.90)	0.995 (0.993–0.997)	
41–50	2408 (23.95)	0.989 (0.987–0.991)	
51–60	1905 (18.94)	0.983 (0.980–0.986)	
61–70	1411 (14.03)	0.970 (0.966–0.973)	
70 +	1226 (12.19)	0.941 (0.933–0.948)	
**Residence**			
Urban	778 (7.74)	0.982 (0.980–0.983)	< 0.001**
Rural	9278 (92.26)	0.979 (0.975–0.983)	
**Education level**			
Primary schools and below	3087 (30.70)	0.962 (0.959–0.966)	< 0.001**
Junior middle school	3797 (37.76)	0.986 (0.984–0.988)	
High school or similar	1904 (18.93)	0.993 (0.990–0.995)	
Junior College and above	1268 (12.58)	0.997 (0.996–0.998)	
A**nnual household income (yuan, RMB)**			
< 50,000	2688 (26.73)	0.959 (0.955–0.964)	< 0.001**
50,000**–**99,999	3316 (32.98)	0.986 (0.984–0.988)	
100,000–149,999	2231 (22.19)	0.992 (0.991–0.994)	
≥ 150,000	1821 (18.11)	0.993 (0.992–0.995)	
**Employment status**			
Retired/homemaker/unemployed/student	2294 (22.81)	0.962 (0.957–0.966)	< 0.001**
Paid employment	7762 (77.19)	0.987 (0.986–0.989)	
**Marital status**^#^			
Married	8464 (84.19)	0.983 (0.981–0.984)	0.017*
Unmarried/divorce/widow	1589 (15.80)	0.975 (0.971–0.979)	
**Regular physical activities**^#^			
Yes	6128 (60.94)	0.984 (0.982–0.985)	< 0.001**
No	3927 (39.06)	0.978 (0.975–0.981)	
**Smoking status**			
Never	7313 (72.72)	0.981 (0.979–0.983)	< 0.001**
Current	2452 (24.38)	0.986 (0.983–0.988)	
Former	291 (2.89)	0.956 (0.942–0.970)	
**Drinking status**			
Never	7958 (79.14)	0.981 (0.979–0.983)	0.193
Current	2098 (20.86)	0.983 (0.981–0.986)	
**BMI **(kg/m^2^)			
≤ 23.9	5641 (56.10)	0.983 (0.982–0.985)	< 0.001**
24.0 to 27.9	3440 (34.21)	0.980 (0.977–0.982)	
≥ 28.0	975 (9.70)	0.975 (0.970–0.980)	
**The number of NCDs**			
0	7638 (75.95)	0.989 (0.988–0.990)	< 0.001**
1	1738 (17.28)	0.972 (0.968–0.976)	
≥ 2	680 (6.76)	0.922 (0.910–0.935)	

**P < 0.001; * *P* < 0.05; # represents 3 missing data on marital status and 1 missing data on physical activity; *CI* confidence interval; *BMI* Body mass index; *NCDs* chronic non-communicable diseases; Paid employment, whether employed, full-time or part-time.

### EQ-5D-5L utility score and EQ-VAS score according to the baseline characteristics

The respondents possessed a mean EQ-5D-5L utility score of 0.981(0.980–0.983) (95%CI) and a mean EQ-VAS score of 83.6(83.2–83.9). Subjects with lower utility scores were those who were elderly, were women, lived in rural areas, were ex-smokers, were unmarried/divorced/widowed, had no paid employment, had high BMI, had a lower education level or lower annual household income, lacked regular physical activity and suffered from one or more NCDs (All *P* < 0.05, Table 1). Similar results were observed for EQ-VAS scores except for sex and drinking (Supplementary Table 2).

### Distribution of EQ-5D-5L utility score and EQ-VAS score

The EQ-5D-5L utility score ranged from − 0.251 to 1, which was left-skewed with the dominant value at 1.00 (i.e., “Full health”). Only 7 subjects had negative utility scores. Of the subjects, 85.6% had the highest utility score (EQ-5D-5L utility score = 1, 11,111 health states). Similarly, the EQ-VAS score ranged from 0 to 100, which was also left-skewed with the major clustering from 80 to 100 (i.e., “the best health you can imagine”). Only 23.6% of subjects had an EQ-VAS score of 90 and above. The other most common health states were 11,121, 11,112, 11,122, and 22,221 in the proportions of 6.4%, 1.3%, 1.2%, and 0.2%, respectively.

### EQ-5D-5L utility score and EQ-VAS score according to health conditions by gender

For men, the condition that had the greatest impact on quality of life was memory-related diseases, followed by stroke and mental diseases, while for women, stroke was the most significant, followed by memory-related diseases and asthma (Table 2). In terms of EQ-VAS scores, slightly different from the utility scores, men with COPD had the lowest VAS scores, while women were still most affected by stroke (Supplementary Table 3).

**Table 2:** EQ-5D-5L utility scores for self-reported diseases with influencing HRQoL in men, women, and the total sample, respectively.

Diseases	Total		Men		Women		*P*
	N	Mean (95%CI)	n	Mean (95%CI)	n	Mean (95%CI)	
No self-reported diseases	6858	0.994 (0.993–0.994)	3205	0.994 (0.993–0.995)	3653	0.993 (0.992–0.994)	0.046*
Hypertension	1815	0.959 (0.953–0.964)	887	0.966 (0.959–0.973)	928	0.952 (0.944–0.959)	< 0.001**
Diabetes mellitus	499	0.951 (0.940–0.961)	209	0.963 (0.949–0.977)	290	0.941 (0.926–0.957)	0.070
Dyslipidemia	325	0.947 (0.934–0.960)	148	0.965 (0.953–0.977)	177	0.932 (0.911–0.952)	0.014*
Stroke	185	0.854 (0.819–0.888)	91	0.868 (0.818–0.918)	94	0.839 (0.791–0.887)	0.038*
CHD	173	0.924 (0.905–0.944)	80	0.934 (0.906–0.962)	93	0.916 (0.888–0.943)	0.173
COPD	114	0.923 (0.894–0.952)	65	0.940 (0.902–0.977)	49	0.901 (0.854–0.948)	0.219
Asthma	77	0.890 (0.841–0.939)	41	0.916 (0.854–0.978)	36	0.861 (0.780–0.941)	0.071
Cancer	110	0.959 (0.938–0.980)	49	0.979 (0.967–0.990)	61	0.943 (0.907–0.980)	0.331
Other respiratory diseases	231	0.935 (0.918–0.952)	135	0.941 (0.918–0.964)	96	0.926 (0.900–0.952)	0.202
Hepatic diseases	63	0.978 (0.967–0.989)	38	0.982 (0.969–0.996)	25	0.972 (0.952–0.992)	0.275
Cardiac diseases	222	0.917 (0.898–0.936)	104	0.927 (0.901–0.954)	118	0.909 (0.881–0.936)	0.196
Kidney diseases	66	0.942 (0.912–0.973)	31	0.955 (0.929–0.981)	35	0.931 (0.877–0.986)	0.903
Digestive system diseases	791	0.946 (0.938–0.954)	349	0.947 (0.935–0.959)	442	0.945 (0.935–0.956)	0.942
Mental diseases	116	0.880 (0.840–0.919)	53	0.872 (0.798–0.947)	63	0.886 (0.844–0.927)	0.311
Memory related diseases	92	0.823 (0.766–0.880)	43	0.795 (0.694–0.896)	49	0.847 (0.783–0.911)	0.833
Arthritis	530	0.921 (0.910–0.933)	209	0.936 (0.921–0.952)	321	0.912 (0.896–0.927)	0.046

**P < 0.001; *P < 0.05; CI indicates confidence interval; *EQ-5D-5L* Euroqol-five dimensions-five levels; *COPD* chronic obstructive pulmonary disease; *CHD* coronary heart disease;

### EQ-5D-5L utility scores for age, education, income, and BMI according to gender

The EQ-5D-5L utility score was presented by age, education level, income, and BMI and was divided according to sex. In general, utility scores are higher in men than in women, with especially significant differences in the 41–50 and 61 + age groups, and the scores for both sexes decline with age. Education level, income and BMI also appeared to influence utility scores in which people with low education, low income and a BMI of 24.0 and above generally scored lower. In addition, we found that men and women had different utility scores in household annual income and BMI (All *P* < 0.05, Table 3).

**Table 3:** EQ-5D-5L norm utility scores according to sex.

Variables	Men (N = 4734)		Women (N = 5322)		*P* value
	N (%)	mean(95%CI)	N (%)	mean(95%CI)	
**Age groups (year)**					
18–30	689 (14.55)	0.994 (0.990–0.998)	718 (13.49)	0.999 (0.998–1.000)	0.056
31–40	769 (16.24)	0.996 (0.994–0.997)	930 (17.47)	0.994 (0.991–0.997)	0.340
41–50	1117 (23.60)	0.990 (0.987–0.994)	1291 (24.26)	0.988 (0.986–0.991)	0.008*
51–60	928 (19.60)	0.984 (0.980–0.988)	977 (18.36)	0.982 (0.979–0.986)	0.177
61–70	656 (13.86)	0.976 (0.971–0.980)	755 (14.19)	0.964 (0.958–0.971)	0.011*
70 +	575 (12.15)	0.947 (0.936–0.958)	651( 12.23)	0.936 (0.926–0.946)	0.015*
**Education level**					
Primary schools or below	1189 (25.12)	0.966 (0.960–0.971)	1898 (35.66)	0.960 (0.955–0.964)	0.063
Junior middle school	1862 (39.33)	0.985 (0.982–0.988)	1935 (36.36)	0.987 (0.985–0.990)	0.805
High school or similar	1064 (22.48)	0.991 (0.988–0.995)	840 (15.78)	0.995 (0.992–0.997)	0.171
Junior college and above	619 (13.07)	0.997 (0.996–0.998)	649 (12.19)	0.998 (0.997–0.999)	0.849
**Annual household income (yuan, RMB)**					
< 50,000	1228 (25.94)	0.962 (0.956–0.969)	1460 (27.43)	0.957 (0.951–0.962)	0.011*
50,000**–**99,999	1521 (32.13)	0.986 (0.984–0.989)	1795 (33.73)	0.985 (0.982–0.988)	0.170
100,000–149,999	1076 (22.73)	0.993 (0.991–0.995)	1155 (21.70)	0.991 (0.989–0.993)	0.092
≥ 150,000	909 (19.20)	0.994 (0.992–0.996)	912 (17.14)	0.992 (0.990–0.994)	0.262
**BMI **(kg/m^2^)					
< 18.5/18.5–23.9	2481 (52.41)	0.984 (0.982–0.987)	3160 (59.38)	0.983 (0.980–0.985)	0.774
24.0–27.9	1782 (37.64)	0.982 (0.978–0.985)	1658 (31.15)	0.978 (0.975–0.981)	< 0.001**
≥ 28.0	471 (9.95)	0.983 (0.976–0.989)	504 (9.47)	0.968 (0.959–0.976)	< 0.001**

**P < 0.001; *P < 0.05; *CI* confidence interval; *BMI* Body mass index.

### EQ-5D-5L utility score and EQ-VAS score according to health problems in each dimension

Utility scores and VAS scores varied significantly according to whether the respondents reported any problems in each dimension. Overall, in terms of the EQ-5D-5L utility score, respondents reporting one problem had a utility score of 0.13 points lower than respondents reporting no problems (All *P* < 0.001, Table 4). Regarding the EQ-VAS, people without problems had 6 scores points higher than people with at least one problem (All *P* < 0.001, Supplementary Table 4).

**Table 4:** EQ-5D-5L utility score by different domains of EQ-5D-5L scale.

Domains	EQ-5D-5L Utility scores	
	Mean (95%CI)	*P* value
**Mobility**		
No problems	0.989 (0.988–0.990)	< 0.001**
Problems	0.662 (0.630–0.694)	
**Self-care**		
No problems	0.987 (0.986–0.988)	< 0.001**
Problems	0.589 (0.541–0.637)	
**Usual activities**		
No problems	0.989 (0.988–0.990)	< 0.001**
Problems	0.677 (0.644–0.709)	
**Pain or discomfort**		
No problems	0.997 (0.996–0.998)	< 0.001**
Problems	0.864 (0.855–0.873)	
**Anxiety or depression**		
No problems	0.990 (0.989–0.990)	< 0.001**
Problems	0.816 (0.799–0.834)	
**All domains**		
Problem-free	1.000	< 0.001**
At least a problem	0.871 (0.863–0.878)	

**P < 0.001; *P < 0.05; *EQ-5D-5L* Euroqol-five dimensions-five levels; *EQ-VAS* European quality of life-Visual Analogue Scale; *CI* confidence interval.

### Health problems reported by respondents

The highest proportion of all respondents reported problems in PD (11.96%), followed by AD (4.68%), while the lowest percentage reported problems in SC (1.34%). The percentage of reported problems with MO, UA, SC, and PD increased with age in the total, male, and female samples, respectively. In contrast, younger age groups (age 18–30 and age 31–40) reported more health restrictions with AD (All *P* < 0.001, Table 5).

**Table 5:** The incidence of self-reported health problems using the EQ-5D-5L descriptive system by age group and sex (%).

Level	MO			SC			UA			PD			AD		
	Total	Men	Women	Total	Men	Women	Total	Men	Women	Total	Men	Women	Total	Men	Women
**All**															
No problems	97.67	97.68	97.67	98.66	98.63	98.68	97.61	97.74	97.50	88.04	89.37	86.87	95.32	95.92	94.78
Problems	2.33	2.32	2.33	1.34	1.37	1.32	2.39	2.26	2.50	11.96	10.63	13.13	4.68	4.08	5.22
**18–30 years**															
No problems	99.79	99.56	100.00	99.72	99.42	100.00	99.50	98.98	100.00	98.36	97.67	99.03	98.29	97.82	98.75
Problems	0.21	0.44	0.00	0.28	0.58	0.00	0.50	1.02	0.00	1.64	2.33	0.97	1.71	2.18	1.25
**31–40 years**															
No problems	99.71	99.74	99.68	99.76	99.87	99.68	99.53	99.61	99.46	97.23	97.14	97.31	96.76	96.74	96.77
Problems	0.29	0.26	0.32	0.24	0.13	0.32	0.47	0.39	0.54	2.77	2.86	2.69	3.24	3.26	3.23
**41–50 years**															
No problems	99.13	98.84	99.38	99.42	99.37	99.46	99.04	98.93	99.15	93.11	94.18	92.18	95.93	97.22	94.81
Problems	0.87	1.16	0.62	0.58	0.63	0.54	0.96	1.07	0.85	6.89	5.82	7.82	4.07	2.78	5.19
**51–60 years**															
No problems	99.00	98.81	99.18	99.48	99.35	99.59	98.90	98.60	99.18	86.61	88.36	84.95	95.49	95.91	95.09
Problems	1.00	1.19	0.82	0.52	0.65	0.41	1.10	1.40	0.82	13.39	11.64	15.05	4.51	4.09	4.91
**61–70 years**															
No problems	96.60	96.95	96.29	98.87	98.93	98.91	96.60	97.41	95.89	76.75	79.73	74.17	93.76	94.36	93.25
Problems	3.40	3.05	3.71	1.13	1.07	1.19	3.40	2.59	4.11	23.25	20.27	25.83	6.24	5.64	6.75
** > 70 years**															
No problems	88.74	89.39	88.17	92.90	93.04	92.78	89.15	90.43	88.02	68.76	72.35	65.59	90.21	91.83	88.79
Problems	11.26	10.61	11.83	7.10	6.96	7.22	10.85	9.57	11.98	31.24	27.65	34.41	9.79	8.17	11.21
*χ*^*2*^	532.89	212.46	324.37	354.58	152.92	203.69	465.89	166.33	307.02	944.75	366.51	581.92	117.15	41.26	81.31
*P*	< 0.001	< 0.001	< 0.001	< 0.001	< 0.001	< 0.001	< 0.001	< 0.001	< 0.001	< 0.001	< 0.001	< 0.001	< 0.001	< 0.001	< 0.001

Values are presented as percentages; *MO* mobility; *SC* self-care; *UA* usual activities; *PD* pain or discomfort; *AD* anxiety or depression.

### Potential influencing factors of HRQoL

Advanced age, living in rural regions, no spouse, quitting smoking, lack of regular physical activity, and suffering from NCDs had a statistically negative impact on HRQoL. In contrast, higher education level, higher annual income, and paid employment exerted a positive effect on HRQoL, as measured by the utility score (All *P* < 0.05, Table 6). The results for the EQ-VAS score were similar, although not statistically significant for physical activity (All *P* < 0.05, Supplementary Table 5).

**Table 6:** Tobit regression analyses on the EQ-5D-5L utility scores.

Variables	Coe	*SE*	*P*
**Sex**			
Male (Ref)			
Female	− 0.006	0.011	0.585
**Age group (year)**			
18–30 (Ref)			
31–40	− 0.054	0.022	0.015*
41–50	− 0.105	0.022	< 0.001**
51–60	− 0.137	0.022	< 0.001**
61–70	− 0.156	0.024	< 0.001**
70 +	− 0.195	0.024	< 0.001**
**Residence**			
Urban (Ref)			
Rural	− 0.078	0.015	< 0.001**
**Education level**			
Primary schools and below (Ref)			
Junior middle school	0.044	0.010	< 0.001**
High school or similar	0.078	0.015	< 0.001**
Junior College and above	0.119	0.023	< 0.001**
**Annual household income (yuan, RMB)**			
≤ 50,000 (Ref)			
50,000**–**99,999	0.041	0.010	< 0.001**
100,000–149,999	0.062	0.013	< 0.001**
≥ 150,000	0.059	0.014	< 0.001**
**Employment status**			
Retired/homemaker/unemployed/student (Ref)			
Paid employment	0.070	0.009	< 0.001**
**Marital status**			
Married (Ref)			
Unmarried/divorce/widow	− 0.040	0.012	0.001**
**Regular physical activities**			
No (Ref)			
Yes	0.028	0.008	0.001**
**Smoking status**			
Never (Ref)			
Current	0.003	0.013	0.837
Former	− 0.065	0.021	0.001*
**Drinking status**			
Never (Ref)			
Current	− 0.005	0.011	0.637
**BMI (kg/m**^2^)			
≤ 23.9 (Ref)			
24.0 to 27.9	− 0.008	0.009	0.340
≥ 28.0	− 0.014	0.013	0.283
**The number of NCDs**			
0 (Ref)			
1	− 0.037	0.010	< 0.001**
≥ 2	− 0.141	0.013	< 0.001**

**P < 0.001; *P < 0.05; *EQ-5D-5L* Euroqol-five dimensions-five levels; *Ref* the reference group; *Coe* coefficient; *SE* Standard error; *BMI* Body mass index; *NCDs* chronic non-communicable diseases; Paid employment, whether employed, full-time or part-time.

### Sensitivity analysis for missing data

Sensitivity analyses were conducted to evaluate the impact of missing values on the EQ-VAS score. The results showed that there was basically no statistical difference between respondents with EQ-VAS scores and all the respondents (Supplementary Table 6). Therefore, the remaining VAS scores could be a good representation of all respondents.

## Discussion

Our study identified some sociodemographic factors influencing HRQoL: old age, lower education levels, lower income levels, residence in rural areas, no spouse, no paid work, lack of regular physical activities and ex-smokers. In addition, NCDs had a significant impact on HRQoL. To our knowledge, this is the first study to estimate the HRQoL population norms for residents in southern Jiangsu Province of China using the EQ-5D-5L questionnaire based on a randomly selected large sample data. The population norms in the study can be used as reference data to compare profiles for patients with specific conditions with data for the average person in the general population in a similar age and gender group^3^ and provide evidence for evaluating the effectiveness of future public health interventions. To date, studies have determined the EQ-5D-5L population criteria of other provinces in China, while few studies have included Jiangsu Province, and those that do include Jiangsu adopt very small and unrepresentative sample sizes^16,26,27^. In addition, previous studies have shown that the EQ-5D-5L scale can effectively reduce the ceiling effect on the 3L scale^28,29^, so the 5L scale is used in this study.

Generally, the mean EQ-5D-5L utility score is 0.98, which is similar to that of the USA (0.97)^30^, slightly higher than that of the urban population in China (0.96)^16^, and significantly higher than those of Poland (0.89)^31^, and Portugal (0.89)^28^. The mean VAS score (83.57) is higher than the national average (80.12) of China^23^. However, a direct comparison of utility scores between different countries or regions is not recommended because regions have different sociodemographic compositions and health policies, which may influence respondents' choice of different dimensions of the EQ-5D scale^32^. The findings showed that the respondents experienced greater problems with PD and AD and fewer problems with SC and UA, which is consistent with the EQ-5D-5L population studies in other countries^5,31,33^. Approximately 12% of participants reported PD, similar to the Chinese average^23^ but well below the averages reported by Poland, the United States, and Greece^34–36^. Interestingly, AD was more common in younger adults (40 years and younger), as has been reported elsewhere^16,26^. One possible explanation is that the younger generation feels more psychological pressure than the older generation because of the fast pace of life in developed cities^16^.

Ageing presents a great challenge to the world, in both developed and developing nations^37^. HRQoL tended to deteriorate with age, as observed in other studies^5,23,31,33,38,39^. As expected, elderly people were more likely to experience problems in all EQ-5D-5L dimensions. The multivariate models established in our study showed that NCDs were a significant predictor of HRQoL. NCDs have become a major cause of death worldwide^40^. People with NCDs had lower utility scores and VAS scores resulting in a worse HRQoL. This is consistent with relevant research results^27,41^.

Previous studies have shown that HRQoL inequality exists in different socioeconomic regions in China, such as Hong Kong (utility score 0.920)^26^ and the urban population in mainland China (0.957)^16,24^. Our study also confirmed this phenomenon: people with higher socioeconomic status (higher income, better education, and paid employment) had better HRQoL, which was consistent with previous studies^23,42,43^. An individual's socioeconomic status is often represented by education, income and employment^23,44^. Educational attainment is the most important of the three factors that constitute an individual's socioeconomic status because it is fairly stable throughout the life course of a person. Furthermore, it can shape one's career and expected income potential. Through this mechanism, its indirect link to health is stronger than its direct impact^45^.

In addition, those without a spouse tended to have a lower HRQoL. These people may experience social isolation and financial stress, which could lower their well-being^46^. People who often engaged in physical activity had relatively better HRQoL. Regularly undertaking both aerobic and muscle-strengthening activities, such as walking and cycling, have significant benefits for health^47^. Our study also found an interesting phenomenon: ex-smokers had significantly lower utility scores. This is similar to the findings of Choi et al., who suggest that there may be a "healthy smoker" phenomenon, where smokers believe that smoking relieves pain and stress, while ex-smokers are likely forced to quit smoking due to a disease^48^.

One advantage of our study is that it has a sufficient sample size (10,056) and a high respondent response rate (98.59%) and is generally representative of the general population in the south of Jiangsu Province. In addition, Tobit regression and GLM were used to replace the traditional linear regression model in the multivariate analysis, which was in line with the distribution characteristics of health utility value and EQ-VAS score, making the analysis results more follow the actual situation. Finally, this study provides utility scores and VAS scores for various diseases, which is rare in related studies^49^ and could be useful for health policymakers when prioritizing resource allocation.

There are some weaknesses worth mentioning in the present study. First, as a cross-sectional design, the correlation between HRQoL and potential variables cannot be interpreted as causal. Moreover, approximately 50% of respondents had missing values for the EQ-VAS. Further analysis indicates that the distribution of all sociodemographic variables for the sample with missing EQ-VAS score values was generally consistent with that for the sample with complete EQ-VAS scores.

## Conclusions

This is the first study to provide HRQoL norms using the EQ-5D-5L scale for adults in southern Jiangsu, China and explore its potential influencing factors. These norm values can be used to rationalize the allocation of limited health resources and to evaluate and compare the effects of different medical interventions in health care. In addition, the study found evident socioeconomic inequalities in HRQoL. Therefore, health inequalities deserve the attention of policymakers, and targeted research on each HRQoL domain can promote further understanding of underlying characteristics of inequalities and identify effective strategies to address them to promote greater equity.

## Supplementary Information

Supplementary Information.
