# Supplementary material for: Estimating population norms for the health-related quality of life of adults in southern Jiangsu Province, China
Source: Sci Rep. 2022 Jun 14;12:9906. doi: 10.1038/s41598-022-13910-x (PMC9198056; doi:10.1038/s41598-022-13910-x)
Supplement: Supplementary file 1 — Supplementary Information. [file 41598_2022_13910_MOESM1_ESM.docx]

**Supplementary materials**

**Supplementary Table 1.** Basic population information of 12 towns (streets) with 17 heath centers in the Liyang city (2020).

**Supplementary Table 2.** EQ-VAS scores according to sample characteristics.

**Supplementary Table 3.** EQ-VAS scores for self-reported diseases with influencing HRQoL in men, women, and the total sample, respectively.

**Supplementary Table 4.** EQ-VAS score by different domains of EQ-5D-5L scale.

**Supplementary Table 5.** Generalized linear model on the EQ-VAS scores.

**Supplementary Table 6.** Sensitivity analysis of EQ-VAS scores.

**Supplementary Figure 1.** Locations of the twelve towns (streets) with seventeen health centers in the Liyang Study.

**Supplementary Table 1.** Basic population information of 12 towns (streets) with 17 heath centers in the Liyang city (2020)

| All towns | Health centers | Permanent population | Region | Residents  (n=764,000, %) | Inclusion participants  (n=10,200, %) |
| --- | --- | --- | --- | --- | --- |
| Shezhu town | Shezhu hospital | 52,021 | Urban | 0 | 0 |
|  |  |  | Rural | 52,021(6.81) | 586(5.75) |
|  | Zhoucheng hospital | 24,668 | Urban | 0 | 0 |
|  |  |  | Rural | 24,668(3.23) | 637(6.25) |
| Nandu town | Nandu hospital | 64,614 | Urban | 0 | 0 |
|  |  |  | Rural | 64,614(8.46) | 625(6.13) |
| Shangxing town | Shangpei hospital | 40,453 | Urban | 0 | 0 |
|  |  |  | Rural | 40,453(5.29) | 613(6.01) |
|  | Shangxing hospital | 38,160 | Urban | 0 | 0 |
|  |  |  | Rural | 38,160(4.99) | 616(6.04) |
| Zhuze town | Zhuze hospital | 59,855 | Urban | 7,622(0.10) | 0 |
|  |  |  | Rural | 52,233(6.84) | 640(6.27) |
| Bieqiao town | Bieqiao hospital | 65,790 | Urban | 0 | 0 |
|  |  |  | Rural | 65,790(8.61) | 626(6.14) |
| Shanghuang town | Shanghuang hospital | 25,776 | Urban | 0 | 0 |
|  |  |  | Rural | 25,776(3.37) | 602(5.90) |
| Daitou town | Daitou hospital | 27,245 | Urban | 0 | 0 |
|  |  |  | Rural | 27,245(3.57) | 600(5.88) |
| Kunlun street | Kunlun hospital | 55,260 | Urban | 38,962(5.10) | 246(2.41) |
|  |  |  | Rural | 16,298(2.13) | 386(3.78) |
| Guxian street | Xinchang hospital | 36,166 | Urban | 0 | 0 |
|  |  |  | Rural | 36,166(4.73) | 612(6.00) |
| Licheng street | Licheng hospital | 87,455 | Urban | 60,221(7.88) | 102(1.00) |
|  |  |  | Rural | 27,234(3.56) | 341(3.34) |
|  | Qingan hospital | 32,372 | Urban | 30,032(3.93) | 292(2.86) |
|  |  |  | Rural | 2,340(0.31) | 301(2.95) |
|  | Madian hospital | 40,592 | Urban | 14,775(1.93) | 222(2.18) |
|  |  |  | Rural | 25,817(3.38) | 297(2.91) |
| Tianmuhu town | Tianmuhu hospital | 40,891 | Urban | 3,328(0.44) | 0 |
|  |  |  | Rural | 37,563(4.92) | 625(6.13) |
|  | Pingqiao hospital | 20,799 | Urban | 0 | 0 |
|  |  |  | Rural | 20,799(2.72) | 598(5.86) |
| Daibu town | Daibu hospital | 51,883 | Urban | 0 | 0 |
|  |  |  | Rural | 51,883(6.79) | 633(6.21) |
| Total | Liyang City/Liyang Study | 76,4000/10,200 | Urban | 154,940(20.28) | 822(8.06) |
|  |  |  | Rural | 609,060(79.72) | 9,378(91.94) |

**Supplementary Table 2.** EQ-VAS scores according to sample characteristics.

| Subject characteristics | EQ-VAS scores | | |
| --- | --- | --- | --- |
|  | n（%） | Mean（95%CI） | *P* |
| Overall | 5080^*^ | 83.57（83.21-83.93） |  |
| Sex |  |  |  |
| Male | 2328（45.83） | 83.71（83.17-84.24） | 0.215 |
| Female | 2752（54.17） | 83.45（82.97-83.94） |  |
| Age group（year） |  |  |  |
| 18-30 | 643（12.66） | 87.28（86.29-88.26） | <0.001** |
| 31-40 | 777（15.30） | 85.94（85.00-86.87） |  |
| 41-50 | 1178（23.19） | 85.22（84.54-85.90） |  |
| 51-60 | 990（19.49） | 83.33（82.54-84.12） |  |
| 61-70 | 789（15.53） | 80.89（79.97-81.80） |  |
| 70+ | 703（13.84） | 78.14（77.15-79.13） |  |
| Residence |  |  |  |
| Urban | 402（7.91） | 87.71（87.03-88.39） | <0.001** |
| Rural | 4678（92.09） | 82.82（82.42-83.22） |  |
| Education level |  |  |  |
| Primary schools and below | 1566（30.83） | 79.92（79.25-80.58） | <0.001** |
| Junior middle school | 1821（35.85） | 84.72（84.17-85.28） |  |
| High school or similar | 985（19.39） | 85.31（84.50-86.12） |  |
| Junior College and above | 708（13.94） | 86.25（85.27-87.24） |  |
| Annual household income（yuan, RMB） |  |  |  |
| <50000 | 1290（25.39） | 79.87（79.14-80.60） | <0.001** |
| 50000**-**99999 | 1510（29.72） | 85.56（85.06-86.07） |  |
| 100000-149999 | 1109（21.83） | 87.35（86.73-87.97） |  |
| ≥150000 | 1171（2305） | 81.49（80.54-82.44） |  |
| Employment status |  |  |  |
| Retired/homemaker/unemployed/student | 1195（23.52） | 82.91（82.15-83.66） | 0.013* |
| Paid employment | 3885（76.48） | 83.77（83.36-84.18） |  |
| Marital status |  |  |  |
| Married | 4406（86.76） | 83.83（83.45-84.21） | 0.002* |
| Unmarried/divorce/widow | 674（13.24） | 81.85（80.76-82.93） |  |
| Regular physical activities |  |  |  |
| Yes | 2369（46.64） | 83.64（83.15-84.13） | 0.019* |
| No | 2710（53.36） | 83.50（82.98-84.03） |  |
| Smoking status  Smoking status |  |  |  |
| Never | 3734（73.50） | 83.87（83.45-84.28） | <0.001** |
| Current | 1184（23.31） | 83.23（82.48-83.98） |  |
| Former | 162（3.19） | 79.19（77.00-81.38） |  |
| Drinking status |  |  |  |
| Never | 3939（77.54） | 84.05（83.65-84.44） | <0.001** |
| Current | 1141（22.46） | 81.92（81.11-82.74） |  |
| BMI（kg/m^2^） |  |  |  |
| ≤23.9 | 2873（56.56） | 84.09（83.62-84.57） | <0.001** |
| 24.0 to 27.9 | 1722（33.90） | 83.09（82.46-83.72） |  |
| ≥28.0 | 485（9.55） | 82.16（81.01-83.31） |  |
| The number of NCDs |  |  |  |
| 0 | 3806（74.92） | 84.64（84.23-85.06） | <0.001** |
| 1 | 927（18.25） | 81.23（80.42-82.04） |  |
| ≥2 | 347（6.83） | 77.60（76.10-79.11） |  |

** *P*<0.001; * *P*< 0.05; The sample size of EQ-VAS was missing 4976; CI, confidence interval; BMI, Body mass index; NCDs, chronic non-communicable diseases; Paid employment, whether employed, full-time or part-time.

**Supplementary Table 3.** EQ-VAS scores for self-reported diseases with influencing HRQoL in men, women, and the total sample, respectively.

| Diseases | Total | | Men | | Women | | *P* |
| --- | --- | --- | --- | --- | --- | --- | --- |
|  | N | Mean（95%CI） | n | Mean（95%CI） | n | Mean（95%CI） |  |
| No self-reported diseases | 3433 | 85.10（84.67-85.52） | 1557 | 85.27（84.64-85.91） | 1876 | 84.95（84.38-85.53） | 0.214 |
| Hypertension | 956 | 80.22（79.38-81.06） | 454 | 80.69（79.47-81.90） | 502 | 79.80（78.63-80.96） | 0.239 |
| Diabetes mellitus | 264 | 78.95（77.29-80.61） | 111 | 77.71（74.97-80.45） | 153 | 79.85（77.77-81.93） | 0.411 |
| Dyslipidemia | 201 | 81.11（79.23-82.99） | 81 | 80.58（77.71-83.45） | 120 | 81.47（78.96-83.98） | 0.448 |
| Stroke | 81 | 73.17（69.16-77.19） | 37 | 74.11（69.07-79.15） | 44 | 72.39（66.15-78.62） | 0.970 |
| CHD | 95 | 75.72（73.22-78.21） | 49 | 73.94（70.32-77.56） | 46 | 77.61（74.13-81.09） | 0.178 |
| COPD | 43 | 73.63（68.61-78.65） | 27 | 71.78（65.07-78.49） | 16 | 76.75（68.66-84.84） | 0.389 |
| Asthma | 30 | 75.73（67.95-83.52） | 15 | 76.80（67.04-86.56） | 15 | 74.67（61.25-88.09） | 0.983 |
| Cancer | 51 | 79.10（75.72-82.48） | 17 | 78.35（71.46-85.24） | 34 | 79.47（75.47-83.47） | 0.879 |
| Other respiratory diseases | 123 | 76.46（73.68-79.23） | 68 | 76.37（72.90-79.83） | 55 | 76.56（71.94-81.18） | 0.672 |
| Hepatic diseases | 39 | 84.56（81.07-88.05） | 20 | 87.25（83.35-91.15） | 19 | 81.74（75.73-87.74） | 0.166 |
| Cardiac diseases | 125 | 78.27（76.00-80.55） | 64 | 77.02（73.84-80.19） | 61 | 79.59（76.27-82.91） | 0.310 |
| Kidney diseases | 40 | 88.33（85.63-91.02） | 21 | 91.00（87.92-94.08） | 19 | 85.37（80.92-89.82） | 0.048* |
| Digestive system diseases | 376 | 79.62（78.38-80.87） | 175 | 80.12（78.25-81.99） | 201 | 79.19（77.51-80.87） | 0.295 |
| Mental diseases | 40 | 78.43（73.80-83.05） | 19 | 78.89（71.61-86.18） | 21 | 78.00（71.53-84.47） | 0.807 |
| Memory-related diseases | 49 | 78.71（74.51-82.92） | 20 | 78.80（72.10-85.50） | 29 | 78.66（72.92-84.40） | 0.894 |
| Arthritis | 303 | 78.01（76.36-79.65） | 121 | 77.00（74.24-79.76） | 182 | 78.68（76.62-80.73） | 0.471 |

** *P*<0.001; * *P*< 0.05; CI indicates confidence interval; EQ-VAS, European quality of life-Visual Analogue Scale; COPD, chronic obstructive pulmonary disease; CHD, coronary heart disease;

**Supplementary Table 4.** EQ-VAS score by different domains of EQ-5D-5L scale

| Domains | EQ- VAS scores | |
| --- | --- | --- |
|  | Mean（95%CI） | *P* value |
| Mobility |  |  |
| No problems | 83.94（83.58-84.29） | <0.001** |
| problems | 67.94（64.83-71.05） |  |
| Self-care |  |  |
| No problems | 83.85（83.49-84.20） | <0.001** |
| problems | 64.41（59.97-68.86） |  |
| Usual activities |  |  |
| No problems | 83.94（83.59-84.30） | <0.001** |
| problems | 67.74（64.50-70.97） |  |
| Pain or discomfort |  |  |
| No problems | 84.37（84.00-84.75） | <0.001** |
| problems | 77.38（76.25-78.50） |  |
| Anxiety or depression |  |  |
| No problems | 83.83（83.46-84.19） | <0.001** |
| problems | 79.09（77.27-80.91） |  |
| All domains |  |  |
| Problem-free | 84.48（84.10-84.85） | <0.001** |
| At least a problem | 78.07（77.06-79.09） |  |

** *P*<0.001; EQ-5D-5L, Euroqol-five dimensions-five levels; EQ-VAS, European quality of life-Visual Analogue Scale; CI, confidence interval.

**Supplementary Table 5.** Generalized linear model on the EQ-VAS scores.

| Variables | Coef. | *SE* | *P* |
| --- | --- | --- | --- |
| Sex |  |  |  |
| Male（Ref） |  |  |  |
| Female | -0.019 | 0.004 | <0.001** |
| Age group（year） |  |  |  |
| 18-30（Ref） |  |  |  |
| 31-40 | -0.037 | 0.007 | <0.001** |
| 41-50 | -0.055 | 0.007 | <0.001** |
| 51-60 | -0.067 | 0.007 | <0.001** |
| 61-70 | -0.079 | 0.008 | <0.001** |
| 70+ | -0.101 | 0.008 | <0.001** |
| Residence |  |  |  |
| Urban（Ref） |  |  |  |
| Rural | -0.053 | 0.005 | <0.001** |
| Education level |  |  |  |
| Primary schools and below（Ref） |  |  |  |
| Junior middle school | 0.025 | 0.005 | <0.001** |
| High school or similar | 0.018 | 0.006 | 0.003* |
| Junior College and above | 0.025 | 0.007 | <0.001** |
| Annual household income（yuan, RMB） |  |  |  |
| ≤50000（Ref） |  |  |  |
| 50000**-**99999 | 0.027 | 0.005 | <0.001** |
| 100000-149999 | 0.029 | 0.005 | <0.001** |
| ≥150000 | 0.048 | 0.006 | <0.001** |
| Employment status |  |  |  |
| Retired/homemaker/unemployed/student（Ref） |  |  |  |
| Paid employment | 0.010 | 0.004 | 0.017* |
| Marital status |  |  |  |
| Married（Ref） |  |  |  |
| Unmarried/divorce/widow | -0.042 | 0.005 | <0.001** |
| Regular physical activities |  |  |  |
| No（Ref） |  |  |  |
| Yes | -0.005 | 0.003 | 0.131 |
| Smoking status  Smoking status |  |  |  |
| Never（Ref） |  |  |  |
| Current | -0.003 | 0.005 | 0.532 |
| Former | -0.033 | 0.010 | <0.001** |
| Drinking status |  |  |  |
| Never（Ref） |  |  |  |
| Current | -0.026 | 0.005 | <0.001** |
| BMI（kg/m^2^） |  |  |  |
| ≤23.9（Ref） |  |  |  |
| 24.0 to 27.9 | -0.006 | 0.003 | 0.106 |
| ≥28.0 | -0.007 | 0.006 | 0.183 |
| The number of NCDs |  |  |  |
| 0（Ref） |  |  |  |
| 1 | -0.010 | 0.005 | 0.027* |
| ≥2 | -0.041 | 0.007 | <0.001** |

** *P*<0.001; * *P*< 0.05; EQ-VAS, European quality of life-Visual Analogue Scale; Ref, the reference group; Coef, coefficient; SE, Standard error; BMI, Body mass index; NCDs, chronic non-communicable diseases; Paid employment, whether employed, full-time or part-time;

**Supplementary Table 6.** Sensitivity analysis of EQ-VAS scores

| Sociodemographic characteristics | Total sample | EQ-VAS samples | *P* |
| --- | --- | --- | --- |
|  | n（%） | n（%） |  |
| Overall | 10056 | 5080^*^ |  |
| Sex |  |  |  |
| Male | 4734（47.08） | 2328（45.83） | 0.146 |
| Female | 5322（52.92） | 2752（54.17） |  |
| Age group（year） |  |  |  |
| 18-30 | 1407（13.99） | 643（12.66） | 0.950 |
| 31-40 | 1699（16.90） | 777（15.30） |  |
| 41-50 | 2408（23.95） | 1178（23.19） |  |
| 51-60 | 1905（18.94） | 990（19.49） |  |
| 61-70 | 1411（14.03） | 789（15.53） |  |
| 70+ | 1226（12.19） | 703（13.84） |  |
| Residence |  |  |  |
| Urban | 778（7.74） | 402（7.91） | 0.702 |
| Rural | 9278（92.26） | 4678（92.09） |  |
| Education level |  |  |  |
| Primary schools and below | 3087（30.70） | 1566（30.83） | 0.211 |
| Junior middle school | 3797（37.76） | 1821（35.85） |  |
| High school or similar | 1904（18.93） | 985（19.39） |  |
| Junior College and above | 1268（12.58） | 708（13.94） |  |
| Annual household income（yuan, RMB） |  |  |  |
| <50000 | 2688（26.73） | 1290（25.39） | 0.992 |
| 50000**-**99999 | 3316（32.98） | 1510（29.72） |  |
| 100000-149999 | 2231（22.19） | 1109（21.83） |  |
| ≥150000 | 1821（18.11） | 1171（2305） |  |
| Employment status |  |  |  |
| Retired/homemaker/unemployed/student | 2294（22.81） | 1195（23.52） | 0.327 |
| Paid employment | 7762（77.19） | 3885（76.48） |  |
| Marital status |  |  |  |
| Married | 8464（84.19） | 4406（86.76） | <0.001 |
| Unmarried/divorce/widow | 1589（15.80） | 674（13.24） |  |
| Regular physical activities |  |  |  |
| Yes | 6128（60.94） | 2369（46.64） | <0.001 |
| No | 3927（39.06） | 2710（53.36） |  |
| Smoking status  Smoking status |  |  |  |
| Never | 7313（72.72） | 3734（73.50） | 0.239 |
| Current | 2452（24.38） | 1184（23.31） |  |
| Former | 291（2.89） | 162（3.19） |  |
| Drinking status |  |  |  |
| Never | 7958（79.14） | 3939（77.54） | 0.024 |
| Current | 2098（20.86） | 1141（22.46） |  |
| BMI（kg/m^2^） |  |  |  |
| ≤23.9 | 5641（56.10） | 2873（56.56） | 0.861 |
| 24.0 to 27.9 | 3440（34.21） | 1722（33.90） |  |
| ≥28.0 | 975（9.70） | 485（9.55） |  |
| The number of NCDs |  |  |  |
| 0 | 7638（75.95） | 3806（74.92） | 0.319 |
| 1 | 1738（17.28） | 927（18.25） |  |
| ≥2 | 680（6.76） | 347（6.83） |  |

Note, EQ-VAS, European quality of life-Visual Analogue Scale; BMI, Body mass index; NCDs, chronic non-communicable diseases; Paid employment, whether employed, full-time or part-time; Statistical analysis was performed based on the chi-square test.


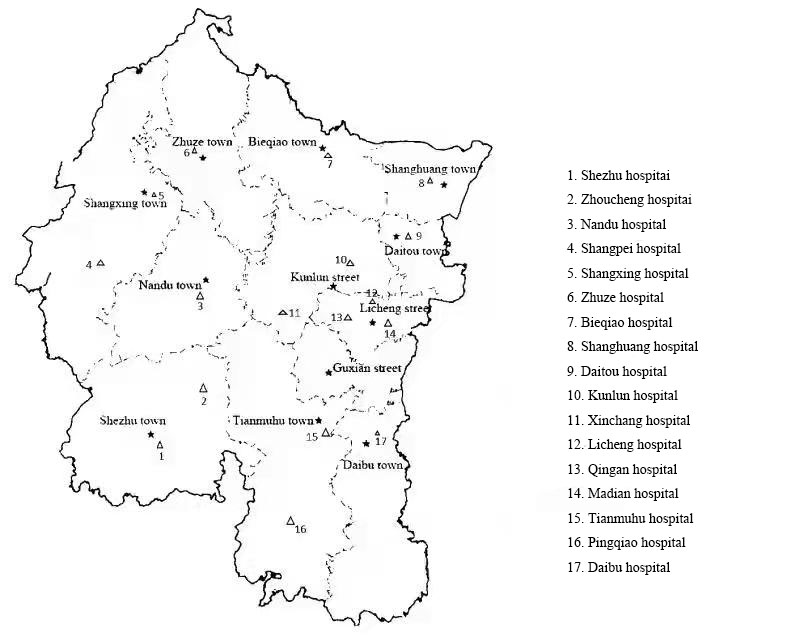


Supplementary Figure 1. Locations of the twelve towns (streets) with seventeen health centers in the Liyang Study.

Note: The Licheng town has three health centers, Tianmuhu, Shangxing and Shezhu town have two heath centers, respectively, and the other 8 towns have only one health centers, respectively.
